# Facile Fabrication of Au Nanoparticles/Tin Oxide/Reduced Graphene Oxide Ternary Nanocomposite and Its High-Performance SF_6_ Decomposition Components Sensing

**DOI:** 10.3389/fchem.2019.00476

**Published:** 2019-07-15

**Authors:** Shoumiao Pi, Xiaoxing Zhang, Hao Cui, Dachang Chen, Guozhi Zhang, Song Xiao, Ju Tang

**Affiliations:** ^1^School of Electrical Engineering, Wuhan University, Wuhan, China; ^2^Hubei Key Laboratory for High-Efficiency Utilization of Solar Energy and Operation Control of Energy Storage System, Hubei University of Technology, Wuhan, China; ^3^State Key Laboratory of Power Transmission Equipment & System Security and New Technology, Chongqing University, Chongqing, China

**Keywords:** rGO, SF_6_ decomposition components, gas sensor, tin oxide, hybrid nanomaterials

## Abstract

A high-performance sensor for detecting SF_6_ decomposition components (H_2_S and SOF_2_) was fabricated via hydrothermal method using Au nanoparticles/tin oxide/reduced graphene oxide (AuNPs-SnO_2_-reduced graphene oxide [rGO]) hybrid nanomaterials. The sensor has gas-sensing properties that responded and recovered rapidly at a relatively low operating temperature. The structure and micromorphology of the prepared materials were characterized by X-ray diffraction (XRD), X-ray photoelectron spectroscopy (XPS), scanning electron microscopy (SEM), Raman spectroscopy, energy-dispersive spectroscopy (EDS), and Brunauer-Emmett-Teller (BET). The gas-sensing properties of AuNPs-SnO_2_-rGO hybrid materials were studied by exposure to target gases. Results showed that AuNPs-SnO_2_-rGO sensors had desirable response/recovery time. Compared with pure rGO (210/452 s, 396/748 s) and SnO_2_/rGO (308/448 s, 302/467 s), the response/recovery time ratios of AuNPs-SnO_2_-rGO sensors for 50 ppm H_2_S and 50 ppm SOF_2_ at 110°C were 26/35 s and 41/68 s, respectively. Furthermore, the two direction-resistance changes of the AuNPs-SnO_2_-rGO sensor when exposed to H_2_S and SOF_2_ gas made this sensor a suitable candidate for selective detection of SF_6_ decomposition components. The enhanced sensing performance can be attributed to the heterojunctions with the highly conductive graphene, SnO_2_ films and Au nanoparticles.

## Introduction

Sulfur hexafluoride is widely used in gas-insulated switchgear (GIS) due to its excellent insulation and arc extinguishing performance (Ma et al., 2016). However, when partial discharge occurs in GIS due to equipment insulation defects, SF_6_ gas decomposes and reacts with trace amounts of water and oxygen present in GIS, thus forming SOF_2_, H_2_S, SO_2_, SO_2_F_2_, and other compounds (Derdouri et al., 1989; Vanbrunt and Herron, 1990; Casanovas et al., 1992; Tang et al., 2013). Studies revealed that the detection of these gas decomposition components can effectively judge the insulation defects to a certain extent, avoid the further development of the insulation defects, and prevent the occurrence of serious insulation accidents (Tang et al., 2012, 2013; Li et al., 2014). Therefore, the development of high-performance detection technology for detecting SF_6_ gas decomposition components has important scientific significance and high application value.

To date, many techniques have been successfully developed to detect SF_6_ decomposition components, such as gas chromatography mass spectrometry technology (Koreh et al., 1997), infrared absorption spectroscopy technology (Kurte et al., 2001), photoacoustic spectroscopy technology (Luo et al., 2015) and gas sensor technology (Zhang et al., 2017). Among these techniques, the gas sensor method has received extensive attention due to its fast reaction speed, simple structure, and low production cost (Dai et al., 2011; Zhang et al., 2013, 2015). As a traditional gas-sensitive material, metal oxide has attracted the attention of many scholars because of its high response, fast response, and recovery speed. Its good sensing properties can be attributed to the unique properties of metal oxides, such as high-surface area to volume ratio, adjustable surface defects, and abundant active substances. To date, many metal oxide-based sensors have been developed to detect SF_6_ decomposition components. For example, Liu et al. used NiO-modified zinc oxide to detect SO_2_, SOF_2_, and SO_2_F_2_ (Liu et al., 2017). Peng et al. used ZnO to detect SOF_2_ (Peng et al., 2013) and Shao et al. used SnO_2_-CuO to detect H_2_S (Shao et al., 2013), which all showed high sensitivity and fast response speed. However, these sensors often operate at high temperatures, resulting in high-power consumption and integration difficulties. Therefore, development of SF_6_ decomposition component detection sensors operating at a relatively low operating temperature has important significance.

Graphene exhibits excellent physical and chemical properties due to its unique two-dimensional structure and electronic properties, such as large specific surface area, extremely large carrier concentration, ultrahigh carrier mobility at room temperature, extremely low electrical noise, and good electron conduction rate (Novoselov, 2004; Ratinac et al., 2010; Chen et al., 2019; Li et al., 2019). Therefore, graphene has always been considered the most promising material for gas detection at a relatively low operating temperature (Schedin et al., 2007; Basu and Bhattacharyya, 2012). However, the intrinsic graphene surface lacks a dangling bond, which is considered as a key to chemisorption. A large number of studies have shown that intrinsic graphene has a good gas-sensitive response to only a few gases, such as NO_2_ and NH_3_ (Nomani et al., 2010; Pumera et al., 2010; Pearce et al., 2011). Subsequently, scholars have confirmed that graphene with certain defects or doping has strong response characteristics to specific gases, and the selectivity and sensitivity of graphene to specific gases can be improved by modifying graphene (Basu and Bhattacharyya, 2012; Gupta Chatterjee et al., 2015; Goutham et al., 2019). Zhang et al. constructed a novel gas sensor based on Au nanoparticles–reduced graphene oxide (rGO), exhibiting an enhanced sensing performance compared with intrinsic graphene for SF_6_ decomposition components (Zhang et al., 2015). However, some disadvantages are observed, such as poor repeatability, long response and recovery time, etc.

To overcome these problems, some scholars have discovered in recent years that the combination of metal oxide and graphene can greatly improve the performance of gas sensors. Many different metal oxides, such as SnO_2_, WO_3_, and Cu_2_O, have been successfully used in rGO modification and exhibited good-sensing properties for gases such as NO_2_ and NH_3_ (Deng et al., 2012; Mao et al., 2012; Kumar et al., 2015). During modification of graphene with metal oxides, SnO_2_ has attracted considerable attention as a typical n-type wide band gap (3.6 eV) metal oxide semiconductor. Wang et al. successfully applied SnO_2_ decorated reduced graphite oxide to detect the SF_6_ decomposition components. The prepared sensor exhibited a response at −3.13% to 10 ppm SOF_2_ at 125°C (Chu et al., 2018). Choi et al. revealed that sensors based on SnO_2_ nanofibers–rGO nanocomposites can detect H_2_S in low concentration at 200°C (Choi et al., 2014). However, the SF_6_ decomposition component sensors mentioned above still have disadvantages, such as long recovery time and relatively high-operating temperature.

Furthermore, studies have found that the addition of noble metals such as Au, Pt, and Ag can greatly improve the sensing performance of gas sensors made of metal oxide and rGO composite films, in which noble metals behave as catalysts and thus the molecules break down into several active substances, which increases gas sensitivity (Kim et al., 2010; Choi et al., 2011; Cui et al., 2012). However, no research has attempted to fabricate sensors based on noble metal/metal oxide/graphene ternary nanocomposites to detect SF_6_ decomposition components.

In this study, we used a hydrothermal synthesis method to prepare a novel sensor based on an AuNPs-SnO_2_ nanoparticles-rGO (AuNPs-SnO_2_-rGO) hybrid to detect the decomposition components of SF_6_ and studied the gas-sensing performance of the sensor in detail. The experimental results showed that the sensor based on AuNPs-SnO_2_-rGO exhibited excellent sensing performance after introduction of AuNPs. The response/recovery time for 50 ppm H_2_S and 50 ppm SOF_2_ at 110°C was (26/35 s, 41/68 s) and was greatly shortened.

## Experimental

### Materials

Stannous chloride dihydrate (SnCl_2_·2H_2_O), polyvinylpyrrolidone (PVP, average molecular mass of 40,000 g/mol), and concentrated hydrochloric acid (HCl, 36–38%), tetrachloroauric acid (HAuCl_4_) were purchased from Sinopharm Chemical Reagent Co., Ltd. (Shanghai, China). Graphene powder was purchased from Nanjing XFNANO Materials TECH Co., Ltd. (Nanjing, China). All reagents purchased were of analytical grade. Deionized water (DIW) was used as the solution in the experiment.

### Preparation of AuNPs-SnO_2_-rGO Nanocomposites

**Figure 1:**
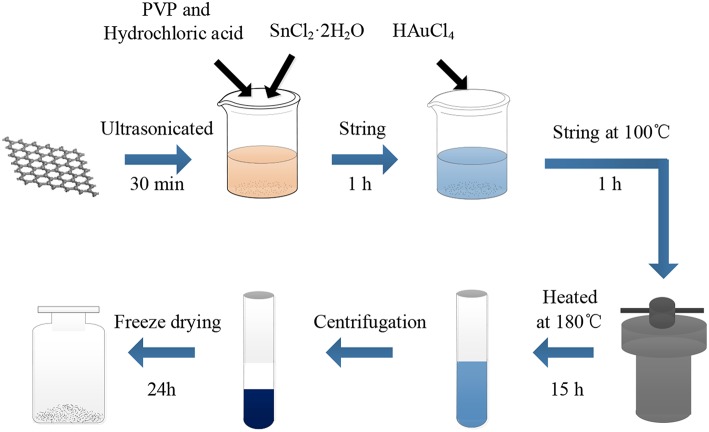
Schematic diagram of the preparation of AuNPs-SnO_2_-RGO hybrid.

The graphene oxide (GO) employed in this study was prepared using a modified Hummer's method (Kovtyukhova et al., 1999). The AuNPs-SnO_2_-rGO nanocomposites were prepared by a method similar to that described by Xu et al. (Zhang et al., 2011). The preparation is shown in Figure 1. In a typical synthesis, 10 mg of GO was sonicated in 50 ml DIW for 30 min. Then, 0.5 ml HCl (36–38%), 100 mg PVP and 500 mg SnCl_2_·2H_2_O were added and stirred for 1 h at room temperature. Next, a certain amount of 0.01 M HAuCl_4_ was added. The experimental solution was stirred at 100°C for 1 h. Then, the solution was placed into a Teflon-lined stainless-steel autoclave to react at 180°C for 15 h. After the autoclave was cooled down to room temperature, the above mixture was washed thrice with absolute ethanol and DIW, followed by freeze-drying for 24 h to prepare AuNPs-SnO_2_-rGO hybrid powder for the subsequent experiment. Four different AuNPs-SnO_2_-rGO samples were obtained at Au ratios of 0.5%, 1.5%, 2.5%, and 5% by weight. Similarly, the rGO, SnO_2_, and SnO_2_-rGO were also prepared.

### Fabrication of Gas Sensors and Testing Method

The prepared material was dispersed in a mixture containing ionic water and isopropyl alcohol at a volume ratio of 1:1 at a concentration of 1 mg/ml and then ultrasonically treated for 30 min. The as-prepared AuNPs-SnO_2_-rGO suspension was then drop-coated with a dropper onto a ceramic plate, which was coated with interdigital electrodes. The size of the interdigitated electrodes with a ceramic plate was 20 mm × 10 mm × 0.635 mm, and both the width and gaps of the 37 pairs of gold tracks on the electrode were 0.1 mm. Subsequently, the prepared sensor was placed in a dry box and dried at 60°C for 24 h to ensure stability before the test. To ensure the uniformity of the sensors prepared in a batch, we made three sensors for the same gas-sensing test, and the results are displayed in the form of reliable average. When the three results fluctuated little, we took the average of them to draw. When the three results fluctuated greatly, we rejected the outliers and retested. Gas sensors based on intrinsic graphene and tin oxide/graphene binary hybrid were prepared in the same manner for comparison.

**Figure 2:**
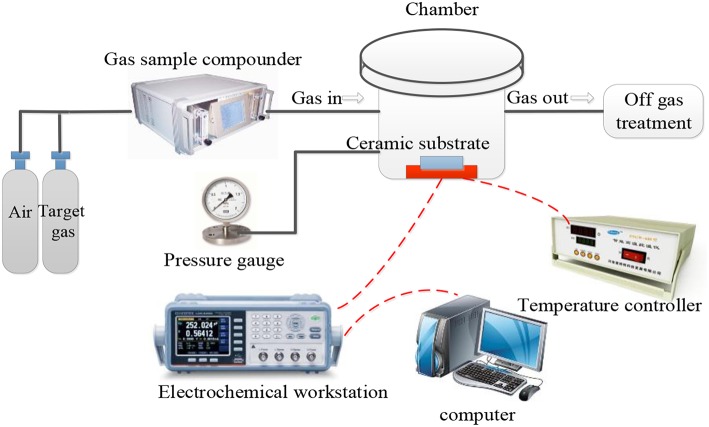
Measurement system for gas sensing experimental.

The gas-sensing test system included the target gas, gas sample compounder, gas-sensing test chamber, electrochemical workstation, and exhaust gas treatment device (Figure 2). The entire experiment was based on a dynamic approach. The sensors were placed in a testing box inside a humidity cyclic chamber (Shanghai Shangqun Technology Co., Ltd.). The temperature in the humidity cyclic chamber was 25 ± 0.5°C. The temperature of the sensor was controlled by a heating electrode on the ceramic substrate. During the experiment, the gas to be tested was introduced through the air inlet and then mixed by the gas-sample compounder. When the sensor was in contact with the incoming gas, the change in resistance throughout the process was recorded by the electrochemical workstation. Due to the corrosive nature of the SF_6_ decomposition components, there may be some changes in sensor parameters after prolonged exposure to these gases (Petrila et al., 2016; Bansode et al., 2017; Manikandan et al., 2018); however, the effects of such variations will not be discussed in this study. The gas flow rate was controlled to 500 ml/min. The resistance measurement interval was 2 s. The test voltage was 50 mV (AC), 1,000 Hz, and the heating voltage was 220 V, 50 Hz. Finally, we calculated the relative change in resistance (sensitivity) of the sensor based on the AuNPs-SnO_2_-rGO hybrid, which is defined as

$$\begin{array}{l} \Delta R/R\left(\%\right)=\frac{100\;\times \;\left(R\;-\;R_{0}\right)}{R_{0}}\times \;100\%, \end{array}$$

where *R* refers to the resistance of the sensor in target gas, and *R*_0_ represents the initial resistance of the sensor in dry air. The response time and recovery time are defined as the time to reach 90% of the total resistance change.

### Instruments

The surface microstructure of the GO, SnO_2_, and AuNPs-SnO_2_-rGO hybrids was investigated by Zeiss SIGMA thermal field-emission scanning electron microscopy and energy dispersive spectroscopy (EDS) of QUANTA200 microscope at a 30 kV acceleration voltage. X-ray photoelectron spectroscopy (XPS) data of the AuNPs-SnO_2_-rGO hybrid were obtained on a Thermo Scientific EscaLab 250Xi spectrometer. X-ray diffraction (XRD) was performed on a Bruker D8 Advance machine by using CuKα radiation (λ = 0.154 nm) with a wide range of scans from 10 to 90° and a speed of 10°/min. Raman spectroscopy data of GO and AuNPs-SnO_2_-rGO hybrids were recorded on a LabRAM HORIBAHR800 Raman spectrometer with a laser with a wavelength of 632.8 nm. Resistance measurements in the experiments were performed using a CHI604E electrochemical workstation. The gas distribution was carried out using a GC500 dynamic gas distributor manufactured by Jiangsu Dong Fang Electric Technology Co., Ltd. (Jiangsu, China).

## Results and Discussion

### Structure and Characterization Analysis

**Figure 3:**
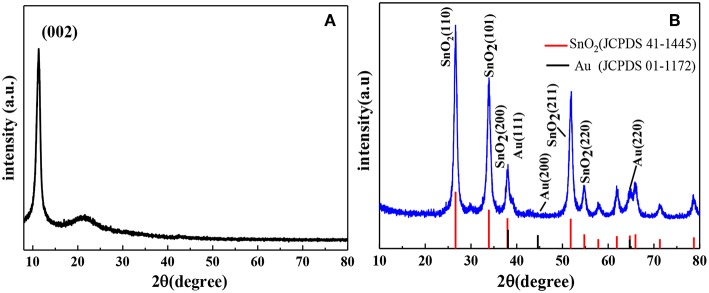
XRD spectra of the samples: **(A)** GO, **(B)** AuNPs-SnO_2_-rGO.

The structure of the AuNPs-SnO_2_-rGO hybrids was examined by the XRD technique. The XRD results of GO and AuNPs-SnO_2_-rGO are shown in Figure 3. Remarkably, GO exhibits a sharp diffraction peak at 2θ of 10.70° due to (002) diffraction of GO, indicating that most of the graphite powder are oxidized to GO (Liu et al., 2010). Figure 3B shows that the XRD pattern of the sample has obvious peak shape and smooth line, indicating that the crystal phase of AuNPs-SnO_2_-rGO is well-formed. The XRD patterns of AuNPs-SnO_2_-rGO nanocomposites reveal four highly diffractive peaks at 2θ of 26.61°, 33.89°, 37.95°, and 51.78° attributed to the (110), (101), (200), and (211) planes of SnO_2_ (JCPDS 41-1445), respectively, indicating the successful formation of SnO_2_ crystals in the composite; this finding is similar to the work reported by Zhang et al. (2014). However, no obvious peak observed in the XRD pattern of AuNPs-SnO_2_-rGO is attributable to Au, which may be due to the low content of Au nanoparticles in the nanocomposite (Zhang et al., 2011).

**Figure 4:**
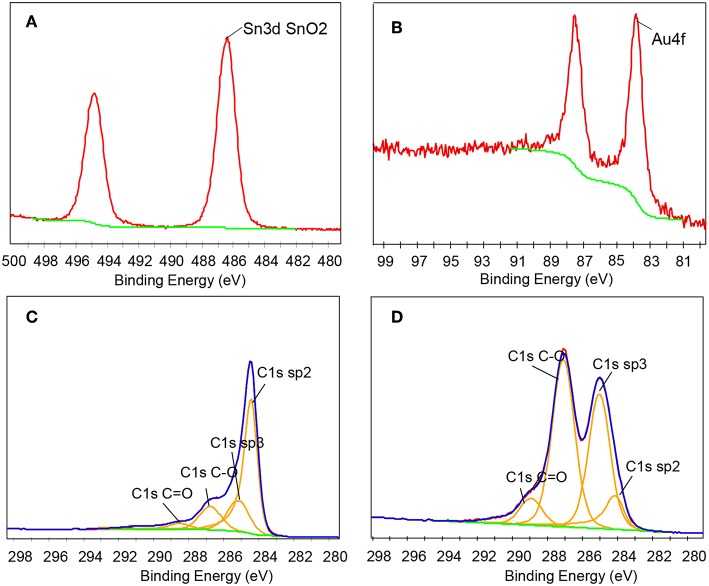
**(A)** Sn3d, **(B)** Au4f, **(C)** C1s XPS spectrum for the AuNPs-SnO_2_-rGO, and **(D)** C1s XPS spectrum for pristine GO.

XPS measurement has proven to be an effective technique for detecting the elemental composition and chemical state of functional materials, especially for rGO-based materials. Therefore, we used XPS technology to characterize the prepared composite. Figure 4A is the Sn3d spectrum of the AuNPs-SnO_2_-rGO ternary nanocomposite. As can be seen from the figure, two strong peaks occur at 486.36 and 494.81 eV, attributed to the binding energy of Sn3d_3/2_ and Sn3d_5/2_, respectively, indicating the SnO_2_ formation (Khlayboonme and Thowladda, 2018; Nguyet et al., 2018). Figure 4B reveals the Au4f spectrum of the AuNPs-SnO_2_-rGO hybrid, confirming the Au presence in the hybrids, with significant signals at 87.6 and 83.36 eV corresponding to metallic Au (Meng et al., 2017). Studies have shown that the C1s XPS spectrum can effectively estimate the chemical reduction level of GO. The C1s spectrum of the AuNPs-SnO_2_-rGO hybrid in Figure 4C has four main parts, including sp^2^ (~284.2 eV), sp^3^ (~284.8 eV), C–O (~286. 7eV), and C=O (~288.5 eV). Sp^2^ reflects the perfect standard structure of graphene, whereas sp^3^ refers to the defects of graphene (Chen et al., 2018). Compared with the C1s spectrum of pristine GO in Figure 4D, the XPS peaks of the oxygen-containing functional groups C-O and C=O are severely weakened. Thus, GO has been successfully reduced in the hydrothermal synthesis.

**Figure 5:**
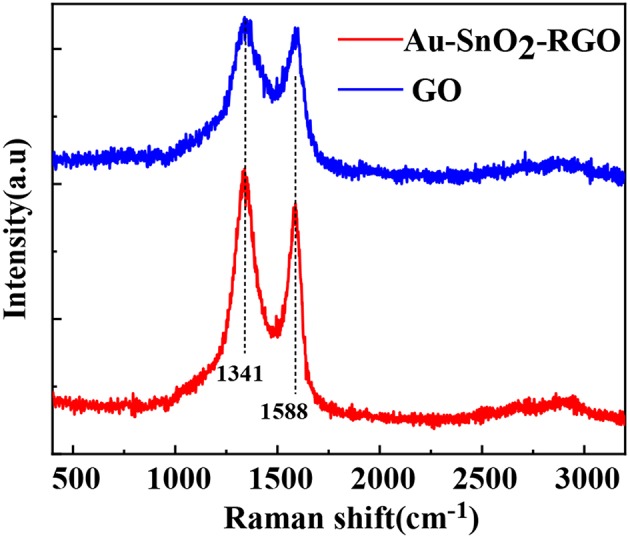
Raman Shift of AuNPs-SnO_2_-rGO and GO.

The Raman spectra of GO and AuNPs-SnO_2_-rGO are shown in Figure 5. Notably, Raman spectroscopy is an effective method for characterizing the structure of graphene substrate materials. Therefore, we further studied the existence of rGO in AuNPs-SnO_2_-rGO nanocomposites by Raman spectroscopy in Figure 5. As reported in other studies, the D peak is mainly caused by the breathing-mode vibration of sp^3^ carbon, which is generally considered the disordered vibration peak of graphene, which is used to characterize the structural defects or edges in graphene samples. This peak occurs at near 1,350 cm^−1^. The G peak is considered the characteristic peak caused by the in-plane vibration of the sp^2^ hybrid carbon atom, which occurs near 1,580 cm^−1^ (Stankovich et al., 2007; Ni et al., 2008; Malard et al., 2009; Dresselhaus et al., 2010). The Raman spectrum of GO in Figure 5 shows two strong peaks at 1,588 and 1,341 cm^−1^, corresponding to well-recorded G and D peaks. Although the Raman spectra of all samples showed G and D peaks, they had some significant differences. Compared with the GO prepared under the same experimental conditions, the intensity ratio of the D band and the G band (ID/IG) of the AuNPs-SnO_2_-rGO ternary is significantly increased, whereas the ID/IG reflects the defect density of graphene materials. The increase in ID/IG indicates the successful GO reduction in the AuNPs-SnO_2_-rGO ternary nanocomposite. In addition, the increase in ID/IG may be due to a decrease in the sp^2^ carbon domain, chemical bonds between the carbon matrix and AuNPs, and defects due to vacancies, grain boundaries and insertion of SnO_2_ nanoparticles. All these observations indicate that we have successfully formed the AuNPs-SnO_2_-rGO ternary hybrid by hydrothermal treatment.

**Figure 6:**
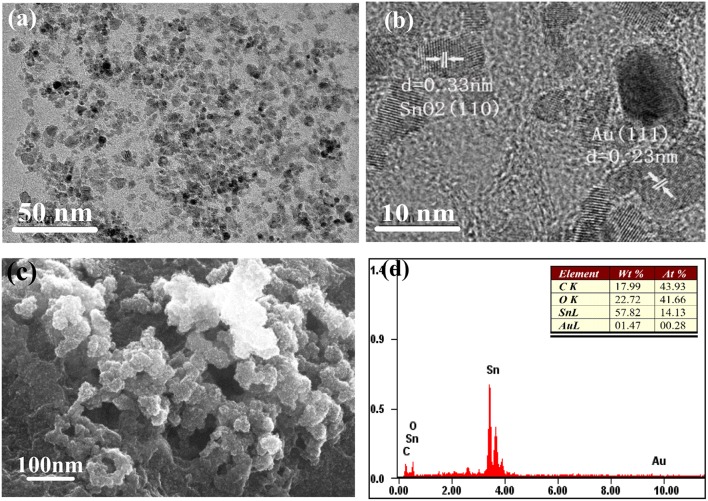
**(a)** TEM, **(b)** HRTEM, **(c)** SEM, and **(d)** EDS of AuNPs-SnO_2_-rGO.

The morphology and structure of the AuNPs-SnO_2_-rGO hybrid was further observed by TEM, HRTEM and SEM as shown in Figure 6. It can be observed from Figure 6a that there are two kinds of nanoparticles with similar particle sizes uniformly dispersed on the surface of RGO. The HRTEM image (Figure 6b) shows an adjacent fringe spacing of 0.235 and 0.33 nm, which correspond to the (111) plane of Au and the (110) plane of SnO_2_ crystal, respectively. The average diameter of the SnO_2_ nanoparticles and the Au nanoparticles is about 5–7 nm. We can clearly see that a large amount of tin dioxide nanoparticles and Au nanoparticles are attached to the pleated rGO surface from a typical SEM image of AuNPs-SnO_2_-rGO hybrid in Figure 6c. The EDS of AuNPs-SnO_2_-rGO products is shown in Figure 6d, in which only Au, C, O, and Sn can be observed. This condition further confirms the successful doping of AuNPs and SnO_2_ nanoparticles on the rGO surface, and the product is quite pure. Combined with XPS analysis, the composition of AuNPs-SnO_2_-rGO hybrids is approximately 1.5% Au, 18% C, 58% Sn, 15.7% O in SnO_2_ and 6.8% O in RGO.

The N_2_ adsorption was performed to explore the architecture of the Au-SnO_2_-RGO composite. The adsorption-desorption isotherms match the type IV based on the apparent hysteresis loops, which indicates the presence of a mesoporous structure (Figure S1). The surface areas of SnO_2_-RGO and Au-SnO_2_-RGO were calculated to be 84.58 and 106.35 m^2^g^−1^, respectively, using the Brunauer–Emmett–Teller (BET) model. It shows an average pore diameter of 3.38 nm for SnO_2_-RGO and 3.29 nm for Au-SnO_2_-RGO as calculated by the Barrett–Joyner–Halenda method (Table S1). The results show that the formation of the ternary composites significantly increases the surface area, which will greatly promote adsorption, thereby increasing the sensitivity of the sensors.

### Sensing Performance of H_2_S and SOF_2_

**Figure 7:**
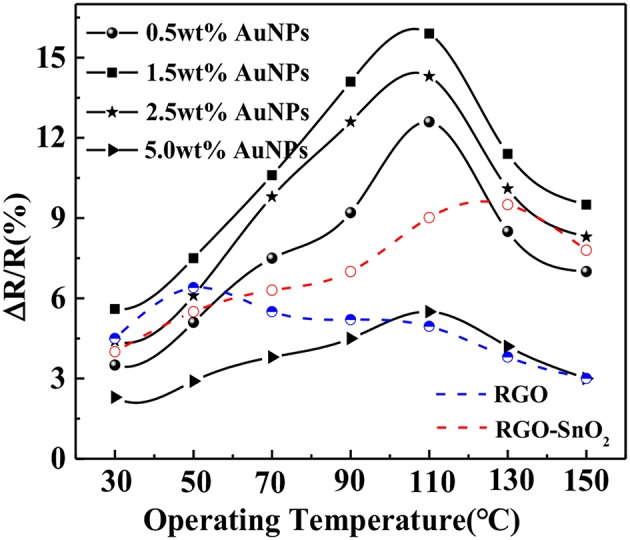
The response of the sensors to 50 ppm SOF_2_ at the temperature of 30–150°C.

We first investigated the effects of the proportion of chloroauric acid and the operating temperature on the sensing performance of the sensors. The sensing performance of all AuNPs-SnO_2_-rGO-based sensors, SnO_2_-rGO-based sensors and rGO-based sensors to 50 ppm SOF_2_ was tested under the operating temperature ranging from 30 to 150°C. The test results are shown in Figure 7. The picture shows that 1.5 wt% AuNPs-SnO_2_-rGO composite exhibited the maximum SOF_2_-sensing response of 15.9% at an optimum operating temperature of 110°C. The optimal operating temperatures for SnO_2_-rGO-based sensors and rGO-based sensors are 130 and 50°C, respectively. When chloroauric acid increased, the sensitivity to SOF_2_ showed a tendency to increase slowly and then decrease. The reason for the analysis may be related to the large nanoparticles of AuNPs when great amount of chloroauric acid is added. For other SF_6_ decomposition gases, the effect of AuNP addition ratio and the optimum operating temperature may be slightly different. However, considering the experiment consistency, we used this 1.5 wt% doping ratio sensor under the optimum operating temperature of 110°C in the next experiment.

From the studies of Nakla et al. (2014) and Shao et al. (2013), we know that SnO_2_ has poor sensing performance at low temperatures. Thus, we only discuss the characteristics of sensors based on intrinsic graphene, SnO_2_-rGO, and AuNPs-SnO_2_-rGO in this study.

**Figure 8:**
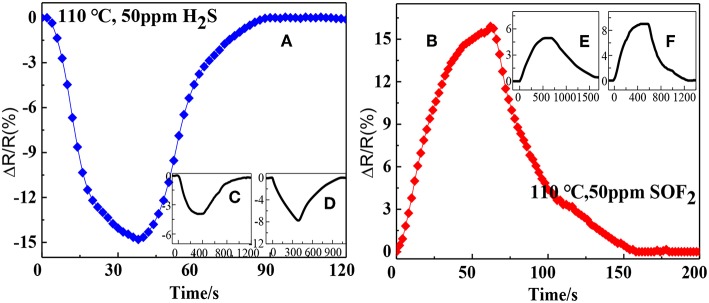
Response recovery curves of the sensor to 50 ppm H_2_S based on **(A)** Au/SnO_2_/RGO, **(C)** RGO, and **(D)** SnO_2_/RGO and the response recovery curves of the sensor to 50 ppm SOF_2_ based on **(B)** Au/ SnO_2_/RGO, **(E)** RGO, and **(F)** SnO_2_/RGO.

Figure 8 illustrates the response and recovery curves of the rGO, SnO_2_-rGO, and AuNPs-SnO_2_-rGO-based sensors to 50 ppm H_2_S and SOF_2_ at 110°C. As can be seen from the figure, the response and recovery speeds are dramatically increased for the AuNPs-SnO_2_-rGO sensor. When exposed to 50 ppm H_2_S, the resistance of AuNPs-SnO_2_-rGO sensor decreases rapidly, and the response, response time, and recovery time are −14.8%, 26 s, and 35 s (Figure 8A), respectively. Conversely, the rGO and SnO_2_-rGO-based sensors show a slow drop in resistance after 50 ppm H_2_S is introduced, with response and recovery times of rGO (210 s, 452 s) and SnO_2_-rGO (308 s, 448 s) of both more than 4 min, respectively (Figures 8C,D). Furthermore, the resistance of the rGO sensor cannot return to its initial values. Interestingly, when exposed to 50 ppm SOF_2_, the rGO, SnO_2_-rGO, and AuNPs-SnO_2_-rGO sensors all showed an increase in resistance, opposite to the H_2_S-sensing behavior. This phenomenon allowed us to distinguish between the two gases. For 50 ppm SOF_2_, the AuNPs-SnO_2_-rGO sensor also has good sensing performance, and the response, response time, and recovery time are 15.9%, 41 s, and 68 s (Figure 8B), respectively. The response, response time, and recovery time of the rGO and SnO_2_-rGO sensors for 50 ppm SOF_2_ are relatively small and long (4.95%, 396 s, and 748 s) and (9.02%, 302 s, and 467 s), (Figures 8E,F). All these observations indicate that the sensor based on AuNPs-SnO_2_-rGO hybrids not only increases the response compared with the sensor based on rGO and SnO_2_-rGO for the detection of H_2_S and SOF_2_. Importantly, its detection response and recovery speed have been greatly improved from 8 min to 60 s.

**Figure 9:**
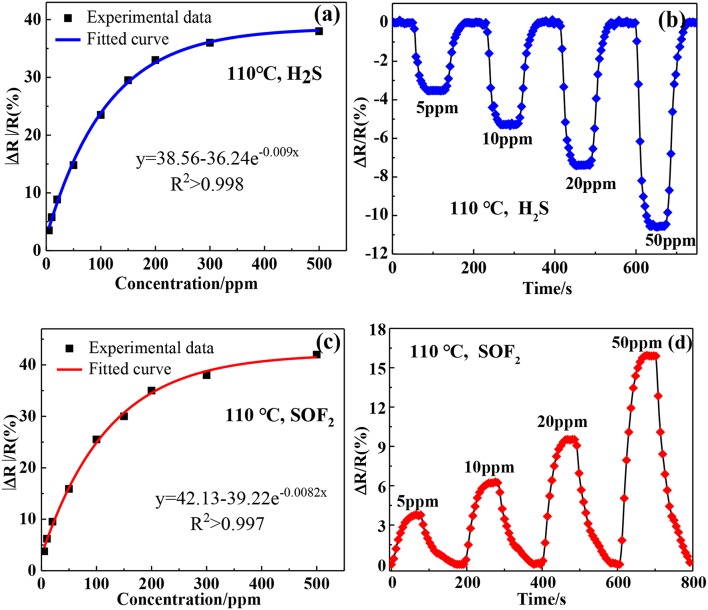
Experimental responses and fitting models toward **(a)** H_2_S and **(c)** SOF_2_ based on Au-SnO_2_-RGO sensor at 110°C. The response-recovery curves of Au-SnO_2_-RGO sensor to 5–50 ppm of **(b)** H_2_S and **(d)** SOF_2_ at 110°C.

Figures 9a,c shows the relationship between the responses of Au-SnO_2_-rGO sensor and gas concentrations at 110°C. Obviously, the response revealed an exponential trend with the increase of the target gas concentration. This result indicated that the adsorption of the target gas by the Au-SnO_2_-rGO sensor almost reached saturation at higher concentrations. Figures 9b,d show that the response values of the Au-SnO_2_-rGO-based sensors are orderly exposed to 5, 10, 20, and 50 ppm H_2_S and SOF_2_ at response values of H_2_S −3.51, −5.78, −8.86, and −14.8% and SOF_2_ of 3.77% 6.21, 9.52, and 15.9%, respectively. Clearly, the Au-SnO_2_-rGO-based sensors respond quickly and reversibly to various concentrations of target gas, indicating that target gas can build a wide concentration detection range of H_2_S and SOF_2_ gas sensors at lower concentrations.

**Figure 10:**
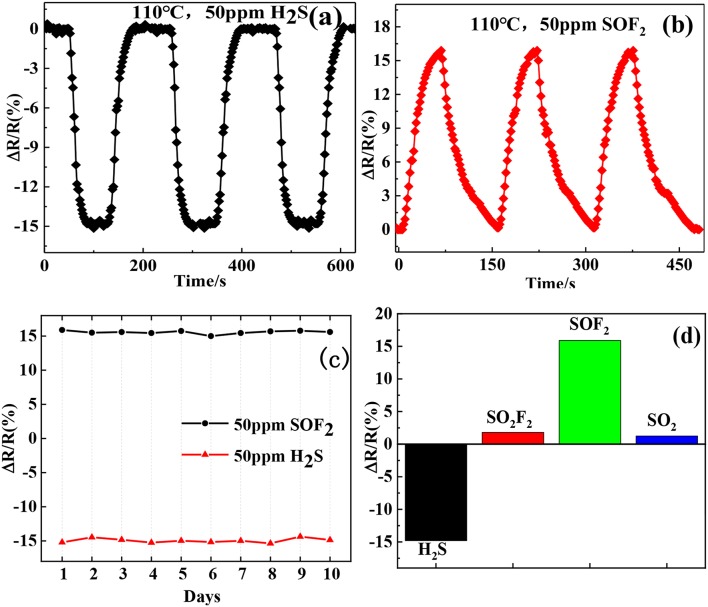
Repeatability curve of Au/SnO_2_/RGO sensor exposed to **(a)** 50 ppm H_2_S and **(b)** 50 ppm SOF_2_; **(c)** the long-term stability of Au/SnO_2_/RGO sensor exposed to 50 ppm H_2_S and SOF_2_. **(d)** selectivity of Au/SnO_−2_/RGO sensor toward 50 ppm H_2_S, SO_2_F_2_, SOF_2_, and SO_2_.

The repeatability and long-term stability of a sensor are important properties for practical applications. We repeatedly exposed the Au-SnO_2_-rGO hybrid sensor to the 50 ppm H_2_S and SOF_2_ gas environment thrice. After each exposure, air was introduced to recover the sensor and then repeated alternately, as shown in Figures 10a,b. We can see clearly that the sensor exhibits a highly consistent response and recovery characteristics in three exposures, demonstrating good repeatability during the cycle test. Next, we conducted a 10-day long-term stability test, and the test results are shown in Figure 10c. The average and relative standard deviations of the response of the Au-SnO_2_-rGO hybrid sensor at a testing concentration of 50 ppm of H_2_S and SOF_2_ for 10 days were (−14.68 and 2.1%) and (15.76 and 1.68%), respectively, exhibiting quite good long-term stability. Furthermore, we also evaluated the selectivity of the Au-SnO_2_-rGO sensor to the typical decomposition components of SF_6_ including H_2_S, SO_2_F_2_, SOF_2_, and SO_2_. Figure 10d represents the sensor response of the Au-SnO_2_-rGO-based sensor for 50 ppm of H_2_S, SO_2_F_2_, SOF_2_, and SO_2_ at 110°C. Interestingly, the Au-SnO_2_-rGO sensor exhibited a large response to H_2_S and SOF_2_ and had minimal response to SO_2_F_2_ and SO_2_. Moreover, when exposed to H_2_S, the sensor displayed a decrease in resistance. However, after the SOF_2_ was turned on, the sensor revealed a rise in resistance. This result allowed us to easily distinguish the two gases by the change in resistance. Therefore, the sensor based on the Au-SnO_2_-rGO hybrid showed good selectivity for both H_2_S and SOF_2_ gases.

**Figure 11:**
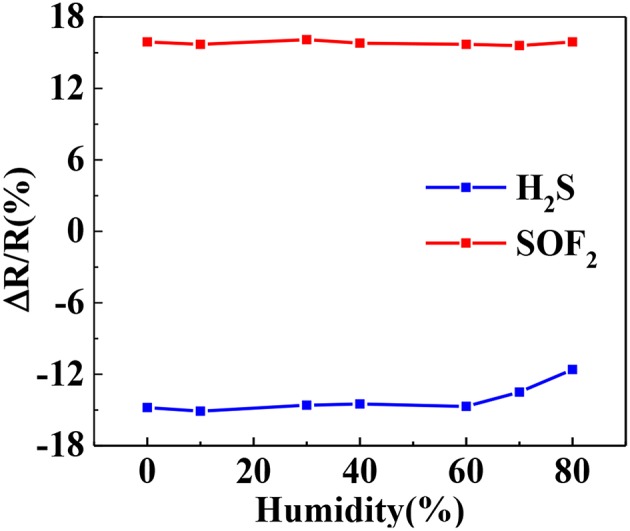
Response values of Au-SnO_2_-rGO to 50 ppm H_2_S and SOF_2_ at different relative humidity. The experiments were performed at 110°C.

Water molecules may compete with target gas molecules during adsorption, thereby affecting gas adsorption. Therefore, the relative humidity effect is an important factor to be considered in sensor testing. Figure 11 illustrates the response curves of the Au-SnO_2_-rGO hybrid to 50 ppm H_2_S and 50 ppm SOF_2_ at different relative humidity. The gas sensor has relatively stable response in the range of 0-60% relative humidity, and the average response value and relative standard deviation are 14.74% (1.56%) and 15.84% (1.06%). However, when relative humidity exceeds 60%, the Au-SnO_2_-rGO sensor's response to H_2_S drops sharply. This result indicates that the Au-SnO_2_-rGO hybrid sensor can operate in the 0–60% relative humidity range.

The sensing performances of the H_2_S and SOF_2_ sensors based on Au-SnO_2_-rGO hybrids were also compared with the previously reported H_2_S and SOF_2_ sensors, as shown in Table 1. ZnO (Peng et al., 2013), CuO–ZnO (Xu et al., 2014), and NiO–ZnO (Liu et al., 2017) sensors responded quickly, but they all worked above 200°C. Although the responses of PPy-WO_3_ (Su and Peng, 2014) and Au-rGO (Zhang et al., 2015) sensors were higher than those of the Au-SnO_2_-rGO sensors, the sensor in this work exhibited short response and recovery times. Compared with Wang et al. (Chu et al., 2018), our sensors can recover to the initial resistance in a very short time and work at relatively low operating temperature. Therefore, the huge advantage of the Au-SnO_2_-rGO sensor was the fast response and recovery at relatively low operating temperature. Importantly, the selectivity of our Au-SnO_2_-rGO sensor allowed for the differentiation of H_2_S and SOF_2_ when exposed to typical SF_6_ decomposition components in GIS. All the above studies showed that our Au-SnO_2_-rGO sensor had great advantages in the detection of SF_6_ decomposition components in GIS.

**Table 1:** Comparison of H_2_S and SOF_2_ sensing performances of our proposed sensor with other published sensors.

**Materials**	**Gas species**	**Concentrations (ppm)**	**Operating temperature**	**Response**	**Response/recovery time(s/s)**	**References**
CuO-ZnO	H_2_S	2	225	1,035%	30/98	Xu et al., 2014
ZnO	SOF_2_	10	300	7%	10/17	Peng et al., 2013
NiO-ZnO	SOF_2_	100	260	22.25	18/22	Liu et al., 2017
PPy-WO_3_	H_2_S	1	RT	81%	360/12600	Su and Peng, 2014
Au-rGO	H_2_S	100	RT	−28.15%	—	Zhang et al., 2015
	SOF_2_	100	RT	23.83%	—	
SnO_2_-rGO	H_2_S	100	125	33.02%	209/900	Chu et al., 2018
	SOF_2_	10	125	−3.24%	255/330	
Au- SnO_2_-rGO	H_2_S	50	110	−14.8%	26/35	This work
	SOF_2_	50	110	15.9%	41/68	

### Gas-Sensing Mechanism

The possible reasons for the superior sensor performance of the sensor are proposed as follows. (i) First, the most important factor for good-sensing performance is the rGO presence. The excellent chemical and physical properties, such as large specific surface area, extremely large carrier concentration, and ultrahigh carrier mobility, provide possibility for the Au-SnO_2_-rGO sensor to detect the H_2_S and SOF_2_ at a relatively low operating temperature. (ii) Second, Au-SnO_2_-rGO is a resistance-type sensor from a macroscopic angle, wherein the principle is based on the conductance variations of the sensing element. The introduction of AuNPs and SnO_2_ increases the electron transport rate of the sensing material, resulting in good sensing performance. (iii) The gas-sensitive response characteristics of the resistance-type sensor are closely related to the surface state of the adsorption substrate. The doping of AuNPs and SnO_2_ significantly increases the number of active sites (such as defect sites and functional groups) on the graphene surface. Subsequently, these increased active sites facilitate gas adsorption and diffusion on the material surface. (iv) The introduction of AuNPs and SnO_2_ results in the formation of new heterojunctions between the various components in the sensing material. In SnO_2_-rGO, the p-n heterojunction formed between SnO_2_ and rGO plays an important role in improving the sensing performance of rGO-based sensors. Scholars have proved that due to the difference in work function between n-type SnO_2_ (4.55 eV) and p-type rGO (4.75 eV), when the two are in contact, the electrons will flow from SnO_2_ to rGO, establishing the electron depletion region (Mao et al., 2012). In ambient air, the formations of chemisorbed ionized oxygen (${\text{O}}_{2}^{-}$) on the surface of SnO_2_ and rGO result in a further alternation in the electron depletion region due to electron transfer from the SnO_2_-rGO to oxygen in a manner similar to that previously reported literature (Lu et al., 2010). When exposed to the target gases (such as H_2_S and SOF_2_), the gas molecules can be easily adsorbed on the active site of the composite and react with the pre-adsorbed oxygen. Thus, the electrons trapped in the chemisorbed-ionized oxygen transfer between the sensing material and gas molecules, resulting in a modification of the electron depletion layer at the SnO_2_ and rGO interfaces. The surface reaction between the H_2_S and SOF_2_ molecules and chemisorbed-ionized oxygen can be proposed as follows (Su and Peng, 2014; Chu et al., 2018):

(1) $$2{\text{H}}_{2}\text{S}+3{\text{O}}_{2}^{-}(\text{ads})\to 2{\text{H}}_{2}\text{O}+2{\text{SO}}_{2}+3{\text{e}}^{-},$$

(2) $$\begin{array}{r} {\text{SOF}}_{2}+{\text{e}}^{-}\to {\text{SOF}}_{2}^{-}, \end{array}$$

(3) $${\text{SOF}}_{2}+{\text{O}}_{2}^{-}{\;}_{\left(\text{ads}\right)}\to {\text{SOF}}_{2}^{-}+{\text{O}}_{2}.$$

In addition, the introduction of AuNPs may lead to the formation of a nano-Sttocky contact between AuNPs and p-type rGO or n-type SnO_2_. AuNPs can dissociate and chemically adsorb O_2_ under ambient air. Therefore, an electron depletion region is formed around the AuNPs by oxygen adsorption. The work function of these regions is higher than the work function of the SnO_2_ nanoparticles (Meng et al., 2018). This condition results in the formation of a nano-Sttocky contact at the Au–SnO_2_ interface and electron transfer from SnO_2_ and rGO to AuNPs. This phenomenon eventually leads to rapid changes in resistance and high sensitivity. (v) Enhanced sensing performance was obtained by the “chemical mechanism” due to the introduction of AuNPs. The “chemical mechanism” proposes that AuNPs activate and decompose molecular oxygen, and its atomic product is then diffused to the SnO_2_ carrier by a spillover effect. This process greatly increases the amount of oxygen that can refill the SnO_2_ surface vacancies and the rate at which this refill occurs, thereby extracting electrons from SnO_2_ quickly at low temperatures (Cui et al., 2012).

**Figure 12:**
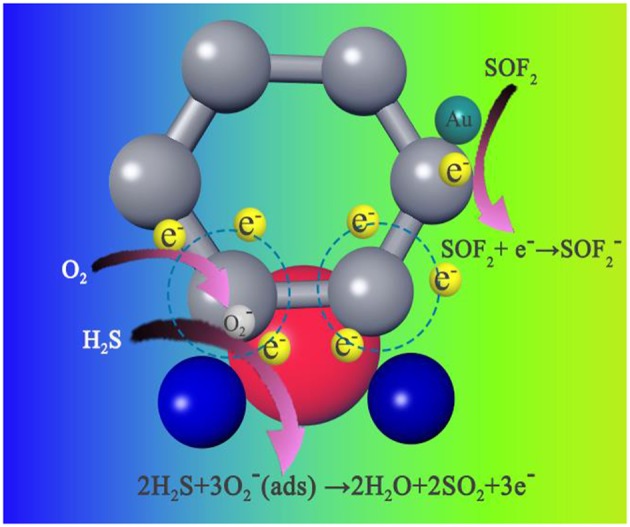
Proposed mechanism for the adsorption behavior of H_2_S and SOF_2_ molecules on Au-SnO_2_-rGO.

In Figure 12, we propose a possible mechanism for the Au-SnO_2_-rGO sensor selectivity to distinguish between H_2_S and SOF_2_. In general, H_2_S is a typical reducing gas (Su and Peng, 2014; Wan et al., 2018). When the Au-SnO_2_-rGO sensor is exposed to H_2_S, electrons trapped by the chemisorbed-ionized oxygen will be released into the conduction band according to formula (1). This phenomenon results in the width of the electron depletion layer to be narrowed, thereby causing a decrease in resistance. By contrast, when exposed to the SOF_2_ gas, the electrons are transferred from the Au-SnO_2_-rGO to the SOF_2_ as shown in formulas (2) and (3). Thus, the electron carrier concentration is decreased, whereby the resistance of the gas sensor is increased.

## Conclusion

In this study, a high-performance sensor for the detection of H_2_S and SOF_2_ has been successfully fabricated by employing Au-SnO_2_-rGO hybrids as sensing materials, which were prepared by hydrothermal synthesis. The gas-sensing properties of AuNPs-SnO_2_-rGO hybrid materials were studied by exposure to target gases. The main conclusions we got are as follows:

1. The introduction of AuNPs in SnO_2_-rGO hybrids significantly improves the sensing properties of the sensors to H_2_S and SOF_2_ at relatively low operating temperature, compared with SnO_2_-rGO hybrids.
2. AuNPs-SnO_2_-rGO sensors had desirable response/recovery time. The response time of Au-SnO_2_-rGO to 50 ppm H_2_S and SOF_2_ at 110°C is (26, 35 s) and (41, 68 s).
3. The sensor has the ability to select four typical decomposition products of SF_6_ and shows the potential to distinguish between H_2_S and SOF_2_ from the direction of resistance change. The AuNPs-SnO_2_-rGO hybrid provides a new sensing material for the manufacture of high-performance SF_6_ decomposition product-detection sensors at a low operating temperature.
4. The enhanced sensing performance can be attributed to the heterojunctions with the highly conductive graphene, SnO_2_ films, and Au nanoparticles.

## Data Availability

All datasets generated for this study are included in the manuscript and/or the Supplementary Files.

## Author Contributions

XZ designed and guided this investigation. SP performed this study and wrote this paper. DC, HC, GZ, SX, and JT implemented the modification of this paper in order to improve its quality. All authors read and approved the final manuscript.

### Conflict of Interest Statement

The authors declare that the research was conducted in the absence of any commercial or financial relationships that could be construed as a potential conflict of interest.
