# Supplementary material for: Facile Fabrication of Au Nanoparticles/Tin Oxide/Reduced Graphene Oxide Ternary Nanocomposite and Its High-Performance SF6 Decomposition Components Sensing
Source: Front Chem. 2019 Jul 15;7:476. doi: 10.3389/fchem.2019.00476 (PMC6660266; doi:10.3389/fchem.2019.00476)
Supplement: Supplementary file 1 [file Table_1.DOCX]

**Supplementary Materials for**

# Facile fabrication of Au nanoparticles/tin oxide /reduced graphene oxide ternary nanocomposite and its high-performance SF_6_ decomposition components sensing

*Xiaoxing Zhang ^1, *^, Shoumiao Pi ^1^, Hao Cui ^2^, Dachang Chen ^1^, Guozhi Zhang^1^, Ju Tang ^1^*

*^1^* *School of Electrical Engineering, Wuhan University, Wuhan 430072, China*

*2 State Key Laboratory of Power Transmission Equipment & System Security and New Technology, Chongqing University, Chongqing 400044, China.*

Fig. S1. N_2_ Adsorption/desorption isotherms for SnO_2_-RGO and Au-SnO_2_-RGO.

Table S1. BET analysis results.

| samples | S_BET_ (m^2^g^-1^) | Average pore diameter(nm) |
| --- | --- | --- |
| SnO_2_-RGO | 84.58 | 3.38 |
| Au-SnO_2_-RGO | 106.35 | 3.29 |
